# Association Between Population Mobility Reductions and New COVID-19 Diagnoses in the United States Along the Urban–Rural Gradient, February–April, 2020

**DOI:** 10.5888/pcd17.200241

**Published:** 2020-10-01

**Authors:** Xiaojiang Li, Abby E. Rudolph, Jeremy Mennis

**Affiliations:** 1 Department of Geography and Urban Studies, Temple University, Philadelphia, Pennsylvania; 2 Department of Epidemiology and Biostatistics, Temple University, Philadelphia, Pennsylvania

**Figure:**
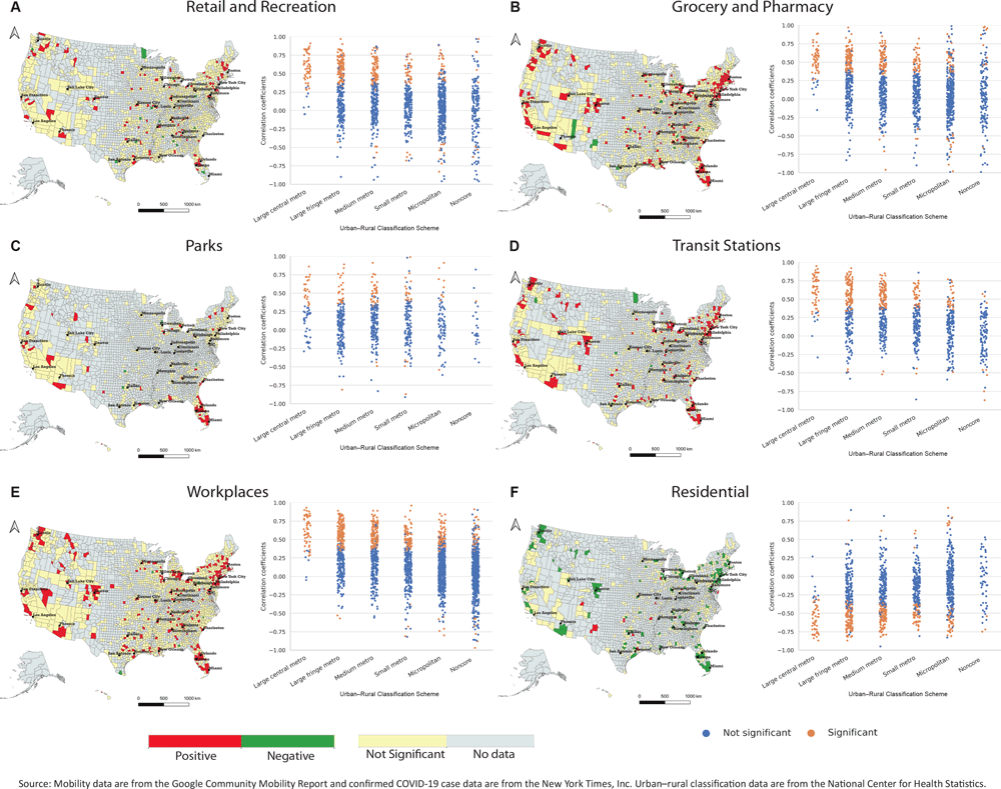
Spatial distribution of the correlation between change in mobility and percentage increase in new COVID-19 cases 11 days later, from February 15 through April 26, 2020, by US county. Correlations are mapped for visits to 6 different types of places and plotted within 6 different urban–rural classifications. Significance is *P* < .05. A decrease in visits to places outside the home, and an increase in time spent at home, are associated with reduced rates of new COVID-19 cases 11 days later in most counties, suggesting that restrictions on mobility can mitigate COVID-19 transmission. The association is stronger in more urban counties, suggesting that mobility restrictions may be most effective in urban areas. Abbreviation: metro, metropolitan.

## Background

As of July 31, 2020, more than 17 million confirmed novel coronavirus disease 2019 (COVID-19) cases had occurred worldwide with more than 668,000 COVID-19–related deaths (1). More than 4.4 million cases and 151,000 deaths occurred in the United States (2). Pre-existing conditions such as asthma and other respiratory conditions, diabetes, and heart disease are associated with COVID-19 illness severity (3), as is race/ethnicity (4), and chronic health problems may persist among survivors (5). Mitigating the COVID-19 pandemic thus has profound implications for chronic disease prevention and outcomes, health disparities, and overall population health.

The basic reproduction number for an infection, R_0_, is influenced by 3 factors: the probability of infection per contact between an infected and a susceptible individual, the average rate of contact between susceptible and infected individuals, and the average duration of infectiousness. In the absence of pharmaceutical interventions, behavioral interventions that reduce contact rates can reduce viral transmission. In response to the COVID-19 pandemic, state and local governments initially required nonessential businesses, schools, places of worship, restaurants, and bars to close; banned large gatherings; and issued stay-at-home directives to promote social (physical) distancing and reduce contact rates. Investigating the relationship between changes in mobility and future changes in the rate of new COVID-19 diagnoses can reveal the effect of these measures on disease transmission (6,7). We mapped the county-level association between changes in population mobility, derived from location histories captured by GPS embedded in mobile phones (8), and the rate of new confirmed COVID-19 cases 11 days later across the United States. We examined the variation across the urban-to-rural gradient, given differences in population density, travel behaviors, the prevalence of COVID-19, and time since the first case was diagnosed in rural versus urban counties (9).

## Data and Methods

County-level daily mobility data for February 15 through April 26, 2020, were obtained from Google’s Community Mobility Report, which comprises aggregated and anonymized data from Google users who turned on the “location history” setting on their cellular telephone (10,11). The data set included 6 location categories, determined by the different types of places encoded within Google Maps: retail and recreation, grocery and pharmacy, parks, transit stations, workplaces, and residential. Daily changes in mobility were measured relative to the median value of travel for the corresponding location type and day of the week from January 3, 2020, through February 6, 2020. County-level daily mobility change was correlated with the daily county growth rate of COVID-19 cases (12) 11 days later (to account for the average incubation period [13]) plus the time delay between testing and state reporting (14), beginning on the day the first confirmed COVID-19 case was reported in each county. A catplot was used to visualize the distribution of the county-level correlation coefficients and their significance for mobility to each location type, stratified by the 6-level urban–rural classification scheme from the National Center for Health Statistics: large central metropolitan, large fringe metropolitan, medium metropolitan, small metropolitan, micropolitan, or noncore county (15). We repeated the analysis by using a 5-day time lag to test the sensitivity of our results.

## Highlights

We plotted the spatial distributions of the correlation coefficients and attendant catplots for each location type. The maps show that retail and recreation, grocery and pharmacy, parks, transit stations, and workplaces generally have significant and positive correlations — a decrease in visits to these locations is associated with a reduced rate of new COVID-19 cases 11 days later. Conversely, an increase in the amount of time spent in residential locations was significantly negatively correlated with an increase in the rate of new COVID-19 diagnoses in most observed counties — staying at home is associated with a slowed growth rate.

Geographic variation is substantial, however, where, in many rural counties, the correlation is not significant. This is illustrated further by the catplots, where for all location types, significant correlations are more likely to occur in urban counties. Indeed, most noncore counties (the most rural) show no significant correlations between change in mobility and the rate of new diagnoses, whereas most large central metropolitan counties show significant correlations for all location types (except parks). Results using the 5-day time lag were consistent with the results presented here.

We acknowledge certain limitations, including extensive missing county mobility data, and that other factors can influence disease transmission and reported cases (eg, testing practices, disease burden, population density, prevalence of chronic health conditions, age distributions, the population living in congregate settings). Additionally, these results reflect cases detected in the United States between February and April, when most states and counties had a combination of stay-at-home directives and business/school closures, and when cases were concentrated in a few urban areas, particularly New York City. In a post-hoc analysis we repeated the analysis by using a February 15 through June 19, 2020, study period. The resulting analogous urban–rural graphs for workplaces and residential places show that the association of mobility reductions with COVID-19 cases we observed for the initial study period dissipates to some extent, particularly in more rural areas (Figure). Notably, May 2020 was a period of decline in COVID-19 cases in the United States; the initial disease hotspots were cooling, and many states began to phase out mobility-reducing directives. This was followed in June by a rapid increase in COVID-19 cases in Florida, Arizona, and other states that did not act aggressively to reduce mobility and encourage wearing masks, with some states reinstating mobility reduction directives in response.

**Figure:**
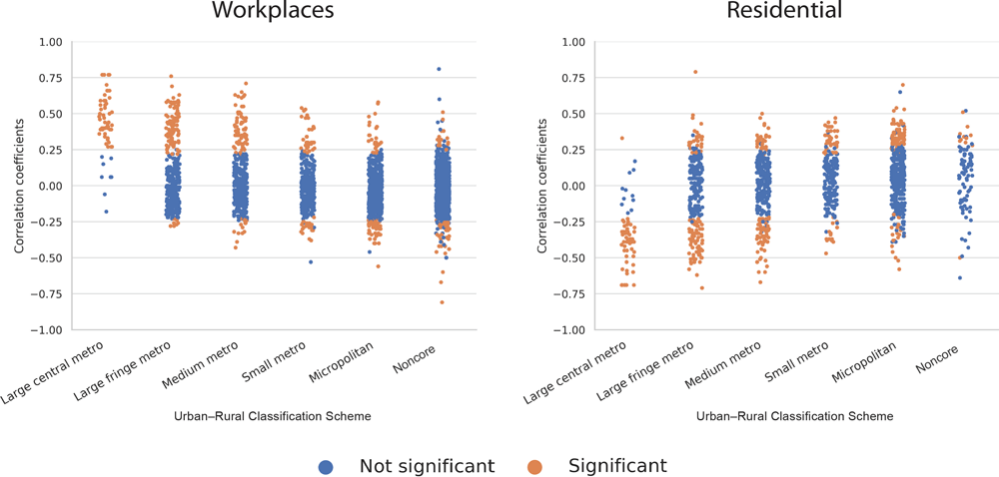
Post-hoc analysis of correlation between change in mobility and percentage increase in new COVID-19 cases 11 days later for February 15 through June 19, 2020, by US county. Correlations are shown for visits to workplaces and residential places and plotted within 6 different urban–rural classifications. Mobility data are from the Google Community Mobility Report, and confirmed COVID-19 case data are from the New York Times, Inc, Urban–rural classification data are from the National Center for Health Statistics. Significance is *P* < .05. The extended study period shows that the association between mobility change and new COVID-19 cases weakened somewhat as compared to the initial study period, particularly in more rural counties, reflecting the changing geographic pattern of disease dynamics occurring in May and June 2020. Abbreviation: metro, metropolitan.

## Action

Although our findings should not be interpreted as a predictive model, these results provide evidence that reductions in population mobility may act to constrain the growth rate in COVID-19 cases, particularly in urban settings, though it is unclear whether the urban–rural differences we observed during the initial rise in COVID-19 cases in the United States will continue in the future, given the changing geography of the pandemic and differences in mitigation approaches used across the country.
